# Analysis of Operator Expertise in MRI/TRUS Fusion-Guided Prostate Biopsy

**DOI:** 10.3390/cancers17233811

**Published:** 2025-11-28

**Authors:** Rouvier Al-Monajjed, Lars Schimmöller, Jale Lakes, Anna Herzum, Anne Hübner, Isabelle Bußhoff, Tim Ullrich, Alexandra Ljimani, Irene Esposito, Peter Albers, Gerald Antoch, Jan Philipp Radtke, Matthias Boschheidgen

**Affiliations:** 1 Department of Urology, Medical Faculty, University Dusseldorf, D-40225 Dusseldorf, Germany; 2 Division of Personalized Early Detection of Prostate Cancer, German Cancer Research Center (DKFZ), D-69120 Heidelberg, Germany; 3 Department of Diagnostic and Interventional Radiology, Medical Faculty, University Dusseldorf, D-40225 Dusseldorf, Germany; 4 Department of Diagnostic, Interventional Radiology and Nuclear Medicine, Marien Hospital Herne, University Hospital of the Ruhr-University Bochum, D-44625 Herne, Germany; 5 Department of Pathology, Medical Faculty, University Dusseldorf, D-40225 Dusseldorf, Germany; 6 Center for Integrated Oncology Aachen Bonn Cologne Düsseldorf (CIO ABCD), D-53127 Bonn, Germany; 7 Department of Radiology, German Cancer Research Center (DKFZ), D-69120 Heidelberg, Germany

**Keywords:** prostate cancer, magnetic resonance imaging, fusion biopsy, learning curve

## Abstract

**Background/Objectives**: This study analyzed the impact of operator experience on the detection of PC and csPC using a standardized MRI/TRUS-fusion biopsy protocol in an experienced high-volume center. **Methods**: Men with mpMRI and subsequent combined TB and SB (2019–2024) using transrectal, software-assisted MRI/TRUS-fusion were retrospectively included. Operators were stratified by experience subgroups (<100 vs. ≥100 procedures). Clinical, MRI, and biopsy data have been assessed. The primary objective was the analysis of the effect of biopsy experience on patient-level PC detection. The secondary objective was the PC detection of PI-RADS and DRE. **Results**: A total of 683 consecutive patients were included (median age 63 years, median PSA 6.5 ng/mL, and median prostate volume 41 mL). Overall, PC and csPC detection were 67% and 51%, with no significant difference in the operator experience subgroups (*p* = 0.63; *p* = 0.23). There were no significant differences for additional csPC detection by SB (7% vs. 5%; *p* = 0.31) or TB (9% vs. 10%; *p* = 0.93) in both subgroups. DRE showed limited diagnostic value (SEN 32%, SPE 88%, PPV 74%, NPV 55%) with no significant variation regarding the experience (*p* = 0.12–1.0). Limitations include a single-center, retrospective design and a lack of a radical prostatectomy specimen. **Conclusions**: In a standardized MRI-targeted biopsy setting, operator experience seems to have a lower influence on PC or csPC detection. High csPC detection in PI-RADS 4–5 supports a TB-only approach, while low rates in PI-RADS 3 suggest follow-up MRI over immediate biopsy. Limited DRE accuracy highlights its declining role in PC assessment.

## Simple Summary

Within a standardized experienced center and a software-assisted fusion biopsy system, less experienced physicians achieved detection rates with transrectal systematic and MRI-targeted cores comparable to more experienced colleagues. These findings suggest that structured fusion biopsy protocols, high-quality imaging, including segmentation/registration, may be more critical than individual experience in ensuring accurate prostate cancer (PC) diagnosis. Suspicious MRI findings (PI-RADS 4–5) need MRI-targeted biopsy, while PI-RADS 3 might receive follow-up MRI first. The digital rectal examination (DRE) showed no significant benefit for PC detection in this cohort.

## 1. Introduction

Multiparametric magnetic resonance imaging (mpMRI) has enhanced prostate cancer (PC) diagnostics by improving detection and characterization of clinically significant PC (csPC; ISUP grade group 2–5) while reducing identification of non-significant PC (nsPC; ISUP grade group 1) [1]. The PRECISION trial showed that mpMRI-targeted biopsy (TB) outperformed transrectal ultrasound-guided systematic biopsy (SB) in detecting csPC and limiting nsPC overdiagnosis [2]. Similar results were found in the MRI-FIRST and 4M studies, highlighting the advantage of mpMRI in biopsy-naïve patients [3,4]. A Cochrane meta-analysis confirmed the MRI pathway’s value, showing a 5% csPC detection increase in biopsy-naïve men and 44% in those with prior negative biopsy [5]. Consequently, mpMRI is now part of international diagnostic guidelines and a standard in PC management [6].

While MRI clearly benefits csPC diagnostics, the optimal use and number of cores for SB and TB remain debated. Ahdoot et al. assessed TB, SB, and combined biopsy in a large single-center study including over 400 radical prostatectomy (RP) specimens [7]. Fusion biopsy showed significantly lower detection of clinically insignificant PC than SB. The combined approach (TB plus SB) detected 9.9% more PC than either alone and upgraded 22% of cases to a higher grade. However, TB alone missed 8.8% of grade group ≥ 3 cancers and underestimated tumor grade in 8.7% [7]. In the RP subgroup, the combined method had fewer upgrades (3.5%) than TB (8.7%) or SB (16.8%) alone [7]. These findings suggest combined biopsy provides higher diagnostic accuracy than TB alone, supported by similar results from Radtke et al. [8]. These findings were further supported by a Cochrane analysis, which demonstrated that TB detects fewer nsPCs compared to SB, while reducing the number of biopsy procedures by approximately one-third [5]. Nevertheless, both TB and SB remain associated with a risk of missing csPC [5]. Two key strategies to enhance csPC detection while limiting low-risk PC diagnosis include optimizing the number of biopsy cores per lesion and accounting for the fusion biopsy learning curve. Ploussard et al. evaluated the impact of PI-RADS classification and cores per lesion on final Gleason grade in RP specimens, recommending at least four cores per lesion [9]. In 2019, the PI-RADS Steering Committee introduced the “target saturation” approach, involving up to nine cores per lesion [10]. Subsequent studies showed this method achieves csPC detection rates comparable to combined TB and SB [11,12,13,14], though it increases detection of ISUP grade 1 PC (5–21%) compared to TB with ≤4 cores [11,13]. Novara et al. [15] reported csPC detection improving from 62% (TB alone) to 72% with four perilesional cores and to 91% with 14 random cores. However, in their study, TB with perilesional cores still missed roughly 30% of csPC, including 15% of contralateral lesions [15].

Conversely, two studies from our group found that taking more than one TB core per lesion provided only marginal improvement in csPC detection and Gleason grade accuracy, in both transrectal and in-bore MRI-guided settings [16,17]. Regarding the learning curve, Checcucci et al. showed that software-assisted fusion biopsy is effective early in training, but reliable sampling, especially for lesions less than 8 mm, requires at least 100 procedures [18]. csPC detection improves with experience, with proficiency typically reached after approx. 100 cases, although definitions of learning curves vary [18,19,20,21]. Missed csPC in TB often results from suboptimal segmentation and inaccurate MRI/US fusion registration [22].

This study examines the impact of operator experience on csPC detection within a highly standardized MRI/TRUS fusion biopsy setting and evaluates the performance of TB and DRE, stratified by experience level.

## 2. Materials and Methods

### 2.1. Study Design and Patient Selection

**Figure 1:**
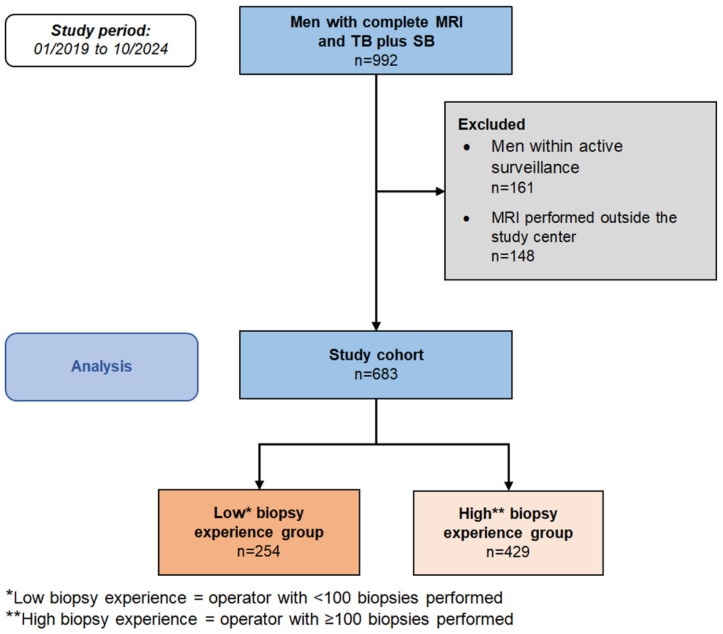
CONSORT Flow Chart.

Between January 2019 and October 2024, this retrospective study included consecutive patients undergoing mpMRI followed by combined TB and SB using MRI/TRUS-fusion biopsy at University Hospital Düsseldorf. Patients under active surveillance for previously diagnosed PC and those with external mpMRI were excluded to ensure imaging standardization. Inclusion details are presented in the CONSORT Flow Diagram (Figure 1). The primary objective was to evaluate the impact of operator experience on patient-level csPC detection across PI-RADS categories. Operators were classified as low (<100) or high (≥100) lifetime MRI/TRUS-fusion biopsies, according to Checcucci et al. [18].

Secondary objectives included assessing the effect of TB core number per lesion on csPC detection, stratified by experience, and comparing TB versus SB detection rates. DRE diagnostic performance was also analyzed by experience level. All mpMRI scans were performed on a 3 Tesla scanner without an endorectal coil, using T2-weighted, diffusion-weighted (DWI), and dynamic contrast-enhanced (DCE) imaging [23]. Lesions were rated per PI-RADS v2.1 by radiologists experienced in prostate imaging (more than 5 years’ experience in reading prostate MRI; M.B.: 6 years, >5000 readings; L.S.: 15 years, >20,000 readings). This study received institutional review board approval.

### 2.2. Biopsy Procedure

Both TB and SB were performed via a transrectal approach. Prior to each procedure, written informed consent was obtained, coagulation status assessed, and a rectal swab collected to guide perioperative antibiotic prophylaxis. Antibiotics, administered orally or intravenously, were selected based on swab microbiology. Patients were placed in the lithotomy position. Preparation included a DRE and rectal cleansing with an antiseptic or anesthetic gel (e.g., lidocaine). Local anesthesia was achieved via TRUS-guided periprostatic nerve block, with 10 mL of 2% lidocaine injected bilaterally adjacent to the neurovascular bundles (5 mL per side). A software-assisted fusion platform (UroNav) was used to align real-time TRUS with prior mpMRI. Lesions were contoured within the software by radiologists. For each mpMRI-detected lesion, in median of two TB cores were taken using an 18-gauge needle and a spring-loaded biopsy device. All patients also received a 12-core SB. TB was always performed prior to SB. Biopsy samples were submitted separately for histopathological evaluation. Resident physicians performing their first fusion-guided biopsies did not receive simulation-based training, but senior supervision was available if needed during initial independent procedures.

### 2.3. Histopathological Analysis and Data Collection

All biopsy specimens were reviewed by experienced genitourinary pathologists at the study site. CsPC was defined as an ISUP grade ≥ 2. Lesions classified as ISUP grade 1 were considered nsPC. Demographic and clinical data, including patient age, PSA levels, PI-RADS scores and histopathological findings, were retrospectively collected. Operator experience and the number of previous fusion biopsy procedures performed were documented for subgroup analysis.

### 2.4. Statistical Analysis

Statistical analyses were conducted using SPSS^®^ Statistics (Version 30, IBM, Ehningen, Germany). Descriptive statistics summarized clinical and mpMRI features. Continuous variables were presented as medians with interquartile ranges (IQR), and categorical variables as absolute counts with percentages. Mann–Whitney U tests were used to compare continuous or ordinal variables between low- and high-experience groups. For patients with multiple mpMRI lesions, the index lesion with the highest PI-RADS score was analyzed. Differences in csPC and nsPC detection by experience level were assessed using Fisher’s exact or Chi-square tests. McNemar tests compared TB and SB performance within the same patients. A *p*-value < 0.05 was considered statistically significant.

## 3. Results

### 3.1. Baseline Characteristics

The final cohort included 683 patients who underwent both TB and SB. A total of 15 operators performed MRI/TRUS fusion biopsies during the study period. The low-experience group comprised 11 operators who had performed <100 lifetime fusion biopsies at the time of the procedure (median of 12; range of 1–99), including urology residents (post-graduate years 1–5) and two board-certified urologists. The high-experience group included 6 operators with ≥100 biopsies (median 301; range 100–535), consisting of board-certified urologists and senior staff surgeons. Due to the longitudinal study time, two operators transitioned from the low- to the high-experience group as additional experience was gained. The low-experience group performed biopsies in 254 patients; the high-experience group in 429 patients. Median age was 63 years (IQR: 53–72), PSA 6.5 ng/mL (IQR: 4.5–10), and MRI-estimated prostate volume 41 mL (IQR: 31–57) (Table 1). No significant differences were observed between experience groups in age, PSA, or prostate volume. Overall, PC and csPC were detected in 67% and 51% of patients, respectively. Detection rates on the patient-level were similar between low- and high-experience groups (PC: 66% vs. 68%, *p* = 0.63; csPC: 48% vs. 53%, *p* = 0.23). Distribution of GG 1–5 (*p* = 0.32–>0.99) and PI-RADS category distribution (*p* = 0.30–0.84) were comparable between groups (Table 1).

### 3.2. PC Detection of MRI (PI-RADS)

For PI-RADS 4, overall PC detection was 68%, with 66% vs. 69% in the respective biopsy experience groups (*p* = 0.69). PI-RADS 5 showed the highest detection rate at 95%, with 97% in low-experience groups and 94% in high-experience groups (*p* = 0.41) (Table 2). CsPC were diagnosed in 46% of PI-RADS 4 (43% vs. 48%, *p* = 0.41), and 84% of PI-RADS 5, with nearly identical detection in both groups (84% vs. 85%, *p* = >0.99). In patients with PI-RADS 3, PC was detected in 27% overall; 25% in the low-experience groups and 29% in the high-experience groups (*p* = 0.76), and csPC in 15% of PI-RADS 3 (13% vs. 16%, *p* = 0.90) (Table 2). In PI-RADS 3, csPC was detected in 15% of cases (95% CI 9–22%). Stratification by PSA density did not meaningfully differentiate cancer risk (PSAD < 0.11: 17% vs. PSAD ≥ 0.11: 15%). Notably, when younger men (age under 55 years) were excluded, PC and csPC detection rates were markedly lower for PI-RADS 3 (PC: 18%; csPC: 9%), and no csPC was detected in men ≥ 55 years within PI-RADS 2 (Supplementary Table S1).

### 3.3. Comparison of SB Versus TB

SB was detected in 11% a csPC, while TB was negative (39/350), with a smaller impact in the high-experience group (9%) compared to the low-experience group (15%), but it was not significantly different (*p* = 0.16). TB alone was identified in 18% a csPC (64/350), with similar rates in the low- and high-experience group (19% vs. 18%; *p* = 0.96). TB was detected in 89% of all csPC overall, with no significant difference by experience (low 85% vs. high 91%, *p* = 0.16). Comparing TB to SB within each group, TB showed higher detection in the total cohort (89% vs. 82%, *p* = 0.02) and the high-experience group (91% vs. 82%, *p* = 0.02), but not in the low-experience group (85% vs. 81%, *p* = 0.53) (Table 3).

SB detected csPC or higher-grade cancer in 21% of cases, with no significant difference between low- (26%) and high- (18%) experience groups (*p* = 0.09). In 46% of cases, SB and TB identified the same grade, with similar rates in low (44%) and high (48%) groups (*p* = 0.60). TB detected csPC or a higher-grade cancer in 33% of patients, with comparable rates between low (30%) and high (34%) experience groups (*p* = 0.44) (Table 4).

The absolute difference in csPC detection in the whole cohort between TB and SB was 7.1% (95% CI 1.5–12.8%), corresponding to an NNB of 14. On multivariable logistic regression analysis including age, PSA density, prostate volume, PI-RADS, PI-QUAL, prior biopsy, calendar year, and operator experience, the operator experience (<100 vs. ≥100) was not associated with csPC detection (OR 1.08, 95% CI 0.82–1.41, *p* = 0.59). Furthermore, to test for a learning effect, we added the cumulative case number as a continuous predictor. This sensitivity analysis also showed no significant association with csPC detection (OR 1.00 per 10 procedures, 95% CI 0.98–1.03, *p* = 0.44), confirming that no incremental learning effect was detectable in our cohort. Also, a subgroup with very low experience within the first 20 biopsies showed no significant differences between SB versus TB PC detection (Supplementary Table S2).

The number of cores per lesion during TB showed that more than one core was required for PC detection in 23% of the lesions. Rates were similar between experience groups: 19% in the low- and 23% in the high-experience group (*p* = 0.32) (Supplementary Table S3).

### 3.4. PC Detection by DRE

In the entire cohort, DRE demonstrated a specificity of 88% and a sensitivity of 32% for csPC detection. The positive predictive value (PPV) was 74%, the negative predictive value (NPV) was 55%, and the overall accuracy was 59%. Performance stratified by operator experience is shown in Table 5 and Table 6, with no significant differences between groups (*p* = 0.12–>0.99).

## 4. Discussion

Our study contributes to the evolving field of MRI-based prostate cancer diagnostics by evaluating the impact of operator experience on MRI-targeted biopsy outcomes in a high-volume center with a standardized biopsy protocol. In our cohort, overall PC detection rates were high (PC 67%; csPC 51%) and consistent across all operator experience levels. Similar detection rates were reported by Novara et al. (PC 61%, csPC 55%) [15], aligning with findings by Klingebiel et al. [24]. Detection rates for PI-RADS 4–5 lesions were particularly high in our study (PC 80%; csPC 62%). Conversely, the low csPC detection in PI-RADS 3 suggests that immediate biopsy may be unnecessary, and follow-up imaging with clinical monitoring may be appropriate. Unlike previous reports proposing specific PSAD thresholds between 0.10 and 0.15 ng/mL/cc for biopsy selection, PSAD did not provide useful risk stratification in our PI-RADS 3 cohort.

Two recent studies help explain the interpretative challenges of PI-RADS 3, which are often scored without confirmed malignancy. In one study, we found that in middle-aged Caucasian men (median age 50) with normal PSA levels, 50% of cases were assigned PI-RADS 3 despite optimal MRI quality. These were largely due to extensive T2-weighted changes and DCE, likely reflecting age-related or inflammatory alterations rather than cancer [25]. A second study showed that MRI interpretation in both younger and older men without PC is complicated by histological features such as prostatitis and atrophy, which increase with higher PI-RADS scores. Notably, younger men often exhibited diffuse hypointense T2w changes despite lower levels of inflammation, suggesting non-malignant histology can significantly influence mpMRI appearance and lead to cancer overestimation [26].

In our study, PC and csPC detection rates were comparable between low- and high-experience operators, suggesting that the learning curve for MRI-targeted biopsy is less critical in standardized settings with radiologist-delineated lesions. This supports the idea that contouring targets and lesion segmentation by radiologists prior to MRI/TRUS-fusion biopsy reduces operator variability. However, even with software-assisted fusion, accuracy may suffer when beginners delineate lesions, as this remains a key challenge in MRI/TRUS-fusion biopsy. Standardized, high-volume fusion workflows using radiologist-delineated lesions and software assistance offer a robust, reproducible diagnostic approach. Our findings show that contouring and lesion segmentation by experienced radiologists can standardize outcomes and reduce the impact of operator experience, with important implications for clinical practice across centers with varying expertise.

Furthermore, SB may be omitted regardless of operator experience. TB alone showed high csPC detection, while SB added limited value, aligning with Ahdoot et al., who reported a 5.9% miss rate with TB alone [7]. This is in contrast to Novara et al., who found a higher increase in csPC detection with added SB [15]. This may be due to the experienced setting and interdisciplinary cooperation between radiology and urology. However, biopsy results were not correlated with RP specimens. This limitation is notable, as Johnson et al. reported that MRI missed 35% of csPC cases [27]. Still, our results support the superiority of TB over SB, consistent with Ahdoot et al., who observed a 13% csPC detection advantage with TB [7]. In our study, TB identified an additional 9.4% of csPC, reinforcing its diagnostic benefit. These findings support omitting SB to streamline protocols without compromising accuracy.

Additionally, two TB cores increased csPC detection, so that the additional diagnostic yield from SB also remained limited. A further effect of additional perilesional cores (e.g., penumbra saturation biopsy) needs to be analyzed, maybe in comparison to standardization and optimization of the TB technique. And, finally, MRI in-bore biopsy may serve as a valuable backup in cases with high MRI suspicion but negative or ISUP 1 findings, potentially improving early csPC detection. Quentin et al. and Ullrich et al. reported that secondary in-bore biopsy detected up to 50% csPC in negative PI-RADS 4–5 cases [28,29].

Our study demonstrates that DRE has limited diagnostic performance across all levels of operator experience, with no statistically significant differences in csPC detection rates between groups. The PROBASE trial also reported low sensitivity of DRE for early-stage prostate cancer, contributing to recommendations against its use in routine screening programs [30]. Similarly, two recent systematic reviews and meta-analyses concluded that DRE lacks sufficient sensitivity and specificity for effective use in primary care and screening settings [31,32]. Taken together, these results suggest that DRE seems an unreliable method for detecting csPC, regardless of operator experience. However, cost-effective DRE might have a higher value in more specific clinical settings (e.g., staging, PSA-negative PC).

Despite these promising results, some limitations should be acknowledged. The retrospective design introduces potential selection bias, and the single-center, tertiary care setting may limit the generalizability of results. Detection rates for PI-RADS 2 and 3 might be overestimated, as only selected cases in these categories underwent biopsy, and a relevant proportion of younger men below 55 years were included. Although radiologist-defined targets helped minimize operator-related variability, the impact of radiologist expertise was not independently evaluated. Still, segmentation by experienced radiologists likely reduces delineation errors, a known factor in MRI/TRUS-fusion biopsy failure. Additionally, the absence of correlation with radical prostatectomy specimens limits the ability to assess potential under-detection of csPC. Finally, all biopsies were performed transrectally in a standardized high-volume workflow, and results may differ in centers with transperineal approaches.

## 5. Conclusions

In a standardized MRI-targeted biopsy setting, detection of csPC using targeted cores of MRI/TRUS-fusion biopsy was not statistically significant between groups of various biopsy experiences. High detection rates in PI-RADS 4–5 support MRI-targeted biopsies, while low rates in PI-RADS 3 favor follow-up MRI over immediate biopsy. The added value of SB was minimal, indicating it may be omitted in high-volume centers. Additionally, DRE showed poor diagnostic performance across all experience levels.

## Figures and Tables

**Table 1:** Baseline characteristics stratified by operator expertise.

		Entire Cohort	Low Biopsy Experience	High Biopsy Experience	*p*-Value
Patients (n)		683	254	429	
**Age** in years; median (IQR)		63 (53.0–72.0)	63 (54.0–73.0)	63 (54.0–71.0)	0.76 *
**PSA** in ng/mL; median (IQR)		6.5 (4.5–10.4)	6.5 (4.4–11.0)	6.6 (4.5–10.3)	0.9 *
**MRI Prostate volume** in ml, median (IQR)		41 (31.0–57.0)	39 (30.0–56.8)	42 (31.0–57.0)	0.12 *
**PI-QUAL v2** (IQR)		3 (3–3)	3 (3–3)	3 (3–3)	>0.99 *
**Number of Cores in TB** (IQR)		2 (2–2)	2 (2–2)	2 (2–2)	>0.99 *
**ISUP GG in Bx** n (%)	1	108 (16%)	45 (18%)	63 (15%)	0.35 ***
	2	147 (22%)	49 (19%)	98 (23%)	0.32 ***
	3	93 (14%)	30 (12%)	63 (15%)	0.35 ***
	4	57 (8%)	21 (8%)	36 (8%)	>0.99 ***
	5	53 (8%)	22 (9%)	31 (7%)	0.60 ***
	1–5	458 (67%)	167 (66%)	291 (68%)	0.63 ***
	2–5	350 (51%)	122 (48%)	228 (53%)	0.23 ***
**PI-RADS** n (%)	2	26 (4%)	9 (4%)	17 (4%)	0.84 **
	3	130 (19%)	53 (21%)	77 (18%)	0.40 ***
	4	302 (44%)	115 (45%)	187 (44%)	0.72 ***
	5	225 (33%)	77 (30%)	148 (35%)	0.30 ***

Statistical tests: * Mann–Whitney U test (MWU), ** Fisher’s exact test, *** Chi-square test. Low biopsy experience < 100 biopsies; high biopsy experience ≥ 100 biopsies.

**Table 2:** Patient-level PC detection rates by PI-RADS stratified by operator expertise.

PI-RADS		Entire Cohort	Low Biopsy Experience	High Biopsy Experience	*p*-Value
**PC**	2	4/26 (15%) **^+^**	3/9 (33%)	1/17 (6%)	0.10 **
	3	35/130 (27%) **^+^**	13/53 (25%)	22/77 (29%)	0.76 ***
	4	205/302 (68%)	76/115 (66%)	129/187 (69%)	0.69 ***
	5	214/225 (95%)	75/77 (97%)	139/148 (94%)	0.41 ***
**csPC**	2	2/26 (8%) **^+^**	1/9 (11%)	1/17 (6%)	>0.99 **
	3	19/130 (15%) **^+^**	7/53 (13%)	12/77 (16%)	0.90 ***
	4	139/302 (46%)	49/115 (43%)	90/187 (48%)	0.41 ***
	5	190/225 (84%)	65/77 (84%)	125/148 (85%)	>0.99 ***

^+^ Inclusion of a relevant proportion of younger men aged 45–55 years. Statistical tests: ** Fisher’s exact test, *** Chi-square test. Low biopsy experience < 100 biopsies; high biopsy experience ≥ 100 biopsies.

**Table 3:** PC and csPC detection of SB and TB for different biopsy experience levels on patient level.

	Entire Cohort	Low Biopsy Experience	High Biopsy Experience	*p*-Value ***
**PC detection (ISUP 1–5)**				
**All PC Cases detected** (n)	458	167	291	
**by SB only** (neg. TB)	59/458 (13%)	29/167 (17%)	30/291 (10%)	**0.04**
**by TB only** (neg. SB)	68/458 (15%)	27/167 (16%)	41/291 (14%)	0.64
**by SB total** (±TB)	390/458 (85%)	140/167 (84%)	250/291 (86%)	0.64
**by TB total** (±SB)	399/458 (87%)	138/167 (83%)	261/291 (90%)	0.04
**TB vs. SB (*p*-value)**	0.48 ****	0.89 ****	0.24 ****	
**csPC detection (ISUP 2–5)**				
**All csPC Cases detected** (n)	350	122	228	
**by SB only** (neg. TB)	39/350 (11%)	18/122 (15%)	21/228 (9%)	0.16
**by TB only** (neg. SB)	64/350 (18%)	23/122 (19%)	41/228 (18%)	0.96
**by SB total** (±TB)	286/350 (82%)	99/122 (81%)	187/228 (82%)	0.96
**by TB total** (±SB)	311/350 (89%)	104/122 (85%)	207/228 (91%)	0.16
**TB vs. SB (*p*-value)**	**0.02** ****	0.53 ****	**0.02** ****	

Statistical tests: *** Chi-square test, **** McNemar Test. Low biopsy experience < 100 biopsies; high biopsy experience ≥ 100 biopsies.

**Table 4:** Patient-level csPC detection of SB versus TB for different biopsy experience levels.

csPC	Entire Cohort	Low Biopsy Experience	High Biopsy Experience	*p*-Value ***
SB > TB	74/350 (21%)	32/122 (26%)	41/228 (18%)	0.09
SB = TB	162/350 (46%)	54/122 (44%)	109/228 (48%)	0.60
TB > SB	114/350 (33%)	36/122 (30%)	78/228 (34%)	0.44

Statistical tests: *** Chi-square test. “>” = detection of PC or higher grade PC. Low biopsy experience < 100 biopsies; high biopsy experience ≥ 100 biopsies.

**Table 5:** Comparison of DRE results.

	Entire Cohort	Low Biopsy Experience	High Biopsy Experience
**DRE positive**			
**all**	n = 151/683 (22%)	n = 46/254 (18%)	n = 105/429 (24%)
**PC**	128 (85%)	39/46 (85%)	89 (85%)
**csPC**	112 (74%)	32/46 (70%)	80 (76%)
**nsPC**	16 (11%)	7/46 (15%)	9 (9%)
**no PC**	23 (15%)	7/46 (15%)	16 (15%)
**DRE negative**			
**all**	n = 532/683 (78%)	n = 208/254 (82%)	n = 324/429 (76%)
**PC**	330/532 (62%)	128/208 (62%)	202/324 (62%)
**csPC**	238/532 (45%)	90/208 (43%)	148/324 (46%)
**nsPC**	92/532 (17%)	38/208 (18%)	54/324 (17%)
**no PC**	202/532 (38%)	80/208 (39%)	122/324 (38%)

Low biopsy experience < 100 biopsies; high biopsy experience ≥ 100 biopsies.

**Table 6:** Sensitivity, specificity, positive and negative predictive value, and accuracy of DRE for csPC detection.

%	Entire Cohort	Low Biopsy Experience	High Biopsy Experience	*p*-Value ***
**Sensitivity (SEN)**	32	26	35	0.12
**Specificity (SPE)**	88	89	87	0.55
**Positive Predictive Value (PPV)**	74	70	75	0.64
**Negative Predictive Value (NPV)**	55	57	54	0.66
**Accuracy (ACC)**	59	59	59	>0.99

Statistical tests: *** Chi-square test. Low biopsy experience < 100 biopsies; high biopsy experience ≥ 100 biopsies.

## Data Availability

The datasets used and/or analyzed during the current study are available from the corresponding author on reasonable request.

## Supplementary Materials

The following supporting information can be downloaded at: https://www.mdpi.com/article/10.3390/cancers17233811/s1, Supplementary Table S1: PC detection in men ≥ 55 years. Supplementary Table S2: results for a very low experience level. Supplementary Table S3: comparison of cases in which more than one core in targeted biopsy (TB) was required to detect a PC, stratified by operator experience and lesion number.

## Abbreviations

The following abbreviations are used in this manuscript:

DRE	Digital rectal examination
PC	Prostate cancer
csPC	Clinically prostate cancer
nsPC	Non-significant prostate cancer
mpMRI	Multiparametric magnetic resonance imaging
PSA	Prostate-specific antigen
PSAD	Prostate-specific antigen density
PI-RADS	Prostate Imaging and Reporting Archiving Data System
PI-QUAL	Prostate Imaging Quality
