# Supplementary material for: Analysis of Operator Expertise in MRI/TRUS Fusion-Guided Prostate Biopsy
Source: Cancers (Basel). 2025 Nov 28;17(23):3811. doi: 10.3390/cancers17233811 (PMC12691296; doi:10.3390/cancers17233811)
Supplement: Supplementary file 1 [file cancers-17-03811-s001.zip › cancers-3941024-supplementary.pdf]

Supplementary Material

Supp. Table S1: Patient-level PC detection in men ≥ 55y.

PI-RADS		N=488
**PC** N=370	2	1/6 (16%)
	3	8/45 (18%)
	4	157/231 (68%)
	5	199/206 (97%)
**csPC** N=289	2	0/6 (0%)
	3	4/45 (9%)
	4	109/231 (47%)
	5	176/206 (85%)

Supp. Table S2: Patient-level results for a very low experience level.

	Very Low Experience level (<20 Bx)	High Experience level (>100 Bx)	p-value <20 vs. >100
**Patients** (n)	103	429	
**PC detection**			
**by SB and TB**	68	291	0.81
**by SB only**	16	30	**0.01**
**by TB only**	14	41	0.30
**by SB total**	54	250	0.32
**by TB total**	52	261	0.07
**csPC detection**			
**by SB and TB**	48	228	0.27
**by SB only**	7	21	0.60
**by TB only**	13	41	0.46
**by SB total**	35	187	0.10
**by TB total**	41	207	0.15

*Statistical tests: ***Chi-square test*
Low biopsy experience <20 biopsies; High biopsy experience ≥100 biopsies

Supp. Table S3: Lesion-level comparison of cases in which more than one core in targeted biopsy (TB) was required to detect a PC, stratified by operator experience and lesion number.

More than one core needed to detect PC	Entire cohort	Low biopsy experience	High biopsy experience	p-value***
**Lesion 1**	87/357 (24.4%)	19/121 (15.7%)	56/232 (24.1%)	0.09
**Lesion 2**	52/252 (20.6%)	19/88 (21.6%)	34/164 (20.7%)	>0.99
**Lesion 3**	32/134 (23.9%)	11/44 (25%)	21/90 (23.3%)	>0.99
**All**	171/743 (23%)	49/253 (19%)	111/486 (23%)	0.32

*Statistical tests: ***Chi-square test*
Low biopsy experience <100 biopsies; High biopsy experience ≥100 biopsies
